# Knowledge, attitudes, and practices regarding melasma among female patients

**DOI:** 10.3389/fpubh.2026.1907193

**Published:** 2026-09-18

**Authors:** Yin Cha

**Affiliations:** Department of Dermatology and Venereology, Dali Bai Autonomous Prefecture People's Hospital, Dali City, Yunnan, China

**Keywords:** health behavior, knowledge, attitude and practice, melasma, photoprotection, structural equation modeling

## Abstract

**Objective:**

Melasma is a common facial hyperpigmentation disorder with substantial psychosocial burden, yet patient-centered knowledge, attitudes, and practices (KAP) remain insufficiently characterized. To investigate the levels of knowledge, attitudes, and practices (KAP) regarding melasma among female patients.

**Methods:**

This cross-sectional study was conducted in Dali, Yunnan Province between June 20, 2024 and November 13, 2025. Data were collected using a self-designed, Chinese-language online questionnaire distributed via the Questionnaire Star (Wenjuanxing) mini-program on WeChat.

**Results:**

A total of 518 valid questionnaires were included. Most participants were aged 31–50 years (67.0%), lived in urban areas (62.9%), and had a bachelor’s degree or above (48.8%). Mean (SD) scores were 8.92 (3.80) for knowledge (possible range: 0–18), 22.97 (5.06) for attitude (8–40), and 20.30 (3.88) for practice (7–35). Structural equation modeling showed that knowledge was directly associated with attitude (*β* = 0.153, *p* = 0.002) and practice (*β* = 0.492, *p* < 0.001), attitude was directly associated with practice (*β* = 0.357, *p* < 0.001), and knowledge was indirectly associated with practice through attitude (*β* = 0.054, *p* = 0.002). Multivariate logistic regression identified rural/suburban residence (OR = 0.546, 95%CI 0.363–0.820) and never using sun protection (OR = 0.384, 95%CI 0.176–0.837) as independent predictors of lower knowledge, whereas prior treatment-seeking predicted higher knowledge (OR = 2.079, 95%CI 1.230–3.515). Each one-point increase in knowledge score (OR = 1.150, 95% CI 1.081–1.223, *p* < 0.001) and each one-point increase in attitude score (OR = 1.071, 95% CI 1.024–1.120, *p* < 0.001) were strong independent predictors of good practice. All three dimension scores were positively correlated (*r* = 0.145–0.390, all *p* ≤ 0.001).

**Conclusion:**

Participants demonstrated knowledge scores below the scale midpoint, attitude scores in the lower half of the possible range, and practices indicating infrequent routine monitoring and active information seeking. Feasible improvements may include structured, photoprotection-centered education with brief standardized counseling and digital follow-up, prioritizing groups with low sun-protection use and rural/suburban residence to improve long-term self-management behaviors.

## Introduction

Melasma is a common acquired disorder of facial hyperpigmentation, presenting as irregular light to dark brown macules and patches on sun-exposed skin, most often involving the cheeks, forehead, and upper lip (1). The condition predominantly affects women of reproductive age and is more frequently observed in individuals with medium to dark skin phototypes (2). Population-based estimates suggest that melasma affects approximately 1% of the general population, whereas prevalence can be considerably higher in certain high-risk settings, reaching up to about 40% among adult women in Southeast Asia (3). Although melasma does not cause direct physical impairment, its visible facial involvement is frequently associated with substantial psychological and social burden (4). Patients often experience appearance-related distress, reduced self-esteem, anxiety, depression, and social avoidance, with quality-of-life impairment comparable to that observed in chronic dermatological conditions involving visible disfigurement, such as psoriasis (1, 5). At the same time, maintaining long-term disease stability in routine clinical practice remains challenging, particularly in the context of variable treatment responses and disease recurrence.

A wide range of therapeutic options is available for melasma, including topical depigmenting agents, oral medications, chemical peels, and laser- or light-based therapies (1, 6). Clinical responses are variable, and relapse is frequently reported, with recurrent hyperpigmentation often observed after treatment discontinuation (7). Effective disease control therefore relies on long-term, integrated management approaches that emphasize strict photoprotection and maintenance therapy to sustain remission (8). Beyond therapeutic limitations, patient management behaviors also influence disease control, as limited understanding of melasma may lead some individuals to delay professional care and rely on non-professional sources, which may pose safety risks and result in adverse cutaneous effects (8, 9). These discrepancies between medical recommendations and everyday practices draw attention to how patients’ knowledge and attitudes influence daily management behaviors.

The Knowledge–Attitude–Practice (KAP) framework offers a structured approach for examining how patients’ understanding of a condition and related perceptions are reflected in everyday self-management behaviors (10). Limited or inaccurate knowledge may give rise to misconceptions or low risk awareness, which can shape attitudes toward prevention and treatment and influence adherence to recommended practices (11). In the melasma field, recent literature has mainly focused on disease mechanisms and therapeutic strategies, with consistent emphasis on photoprotection in prevention and long-term management, whereas comparatively fewer studies have explored patients’ knowledge, attitudes, and practices in a structured manner (6, 12). Consequently, patient-centered investigations that simultaneously assess all three KAP components in melasma remain uncommon, and structured KAP surveys targeting melasma populations particularly in China are still scarce. In contrast, KAP-based studies have been more frequently applied to other chronic dermatologic conditions, such as acne and psoriasis, where patient knowledge and self-management behaviors have been systematically examined. For example, previous surveys have assessed knowledge, attitudes, and practices among patients with acne or psoriasis in different populations, demonstrating the utility of the KAP framework for identifying educational gaps and behavioral barriers in chronic skin diseases (13, 14).

This study therefore applies the KAP framework to Chinese female patients with melasma to examine disease-related knowledge, treatment attitudes, and real-world management practices. By identifying areas where understanding is incomplete or behaviors diverge from clinical recommendations, the findings may inform more targeted patient education and support strategies. Improved alignment between clinical guidance and patient behavior may help reduce inappropriate treatment practices, support sustained disease control, and contribute to better long-term outcomes for individuals living with melasma.

## Materials and methods

### Study design and participants

This cross-sectional study was conducted in Dali, Yunnan Province between June 20, 2024 and November 13, 2025. Female patients diagnosed with melasma were recruited using convenience sampling. The inclusion criteria were as follows: (1) female gender and age ≥18 years with full civil capacity; (2) either self-reported facial melasma or clinically diagnosed with facial melasma by attending medical staff; and (3) ability to understand the questionnaire content and access the survey via smartphone WeChat by scanning a QR code. All participants were required to read the online informed consent form and actively select the “agree to participate” option before proceeding to the formal questionnaire. Participants were excluded if they (1) were unwilling to participate, (2) had a questionnaire completion time of less than 60 s or exceeding 1,800 s, or (3) provided questionnaire data with abnormal values in other items. This study was approved by the Ethics Committee of Dali Bai Autonomous Prefecture People’s Hospital (KS20240827), and all participants provided written informed consent.

### Procedures

A self-designed questionnaire was employed in this study. Its development was informed by relevant clinical guidelines and published literature, and the initial version was refined based on feedback from two experts in dermatology and aesthetic medicine from affiliated institutions—one specializing in laser and aesthetic dermatology, the other in general dermatology and medical aesthetics. Their suggestions focused on expanding the questionnaire to include items related to occupational and environmental exposures, sun exposure duration, family and medical history (particularly thyroid, breast, and gynecological conditions), medication and cosmetic use, anxiety status, patients’ expectations regarding melasma treatment, sun protection practices, and knowledge of skin-lightening products. The questionnaire was then pilot-tested in a small sample to ensure clarity and feasibility.

The final questionnaire was administered in Chinese and comprised four sections. The basic information section included 15 items. The knowledge section consisted of 10 items, of which Item 6 served as a trap question for quality control; the remaining nine items were scored as “very familiar” (2 points), “heard of it” (1 point), or “not sure” (0 points), resulting in a total possible score of 0–18. The attitude section included eight items assessed using a five-point Likert scale ranging from positive to negative responses (5–1 points), with a total score range of 8–40. The practice section comprised seven items and was similarly evaluated using a five-point Likert scale (5–1 points), yielding a total possible score of 7–35. The inclusion of face-washing frequency (P4) was based on the rationale that excessive cleansing (>3 times daily) may compromise the cutaneous barrier and aggravate irritation-related post-inflammatory hyperpigmentation. Dietary restriction items (P5) were included because traditional Chinese dermatology counseling frequently advises limiting stimulants (e.g., coffee) and spicy foods to reduce facial vasodilation, and these self-imposed restrictions represent common real-world behaviors warranting documentation.

The questionnaire was distributed using a convenience sampling approach. Data were collected through an online survey created with the Questionnaire Star (Wenjuanxing) mini-program on WeChat, and participants accessed the survey by scanning a QR code. To ensure data quality and completeness, each IP address was permitted to submit the questionnaire only once, and completion of all items was mandatory. All submitted questionnaires were reviewed by the research team for completeness, internal consistency, and plausibility.

### Sample size calculation

Sample size was calculated using the formula for cross-sectional studies: α = 0.05, $\;n={\left(\frac{Z_{1-\alpha /2}}{\delta }\right)}^{2}\times p\times (1-p)$ where $Z_{1-\alpha /2}$ =1.96 when α = 0.05, the assumed degree of variability of *p* = 0.5 maximizes the required sample size, and δ is admissible error (which was 5% here). The theoretical sample size was 480 which includes an extra 20% to allow for subjects lost during the study.

### Statistical analyses

Statistical analyses were conducted using R software (version 4.3.2) and Stata (version 18.0). Continuous variables were first assessed for normality. Data conforming to a normal distribution are presented as means with standard deviations (SD), whereas non-normally distributed data are summarized as medians with the 25th and 75th percentiles. Categorical variables are expressed as numbers and percentages [n (%)]. For group comparisons of knowledge, attitude, and practice scores, independent-samples t tests were applied to normally distributed continuous variables when comparing two groups, while the Wilcoxon–Mann–Whitney test was used when normality assumptions were not met. Comparisons across three or more groups were performed using analysis of variance (ANOVA) for normally distributed data with homogeneous variances, whereas the Kruskal–Wallis test was employed for non-normally distributed data. Associations among the three dimension scores were examined using the Pearson correlation coefficient for normally distributed variables and the Spearman rank correlation coefficient when normality assumptions were violated. To explore factors associated with each dimension, univariate and multivariate regression analyses were performed with knowledge, attitude, and practice scores treated as dependent variables. For outcome classification, the median value of each dimension score was used as the cutoff point. Given the absence of established clinical cutoff values for KAP dimensions in melasma, the median was chosen as a pragmatic threshold to dichotomize participants into higher and lower score groups, consistent with prior KAP studies where validated cutoffs are unavailable. Variables with a *p* value <0.10 in univariate analyses were subsequently entered into the multivariate models. All *p* values were reported to three decimal places, and a two-sided *p* value <0.05 was considered statistically significant. Within the KAP theoretical framework, structural equation modeling (SEM) was conducted using the lavaan package (version 0.6–16) in R to examine the hypothesized mediating role of attitude in the knowledge–practice relationship and to estimate standardized direct and indirect path coefficients (Std.all). Model fit was evaluated using the root mean square error of approximation (RMSEA), standardized root mean square residual (SRMR), Tucker–Lewis index (TLI), and comparative fit index (CFI), with acceptable fit defined as RMSEA <0.08, SRMR <0.08, TLI > 0.80, and CFI > 0.80.

## Results

### Questionnaire quality

Initially, this study collected a total of 595 questionnaires. The following samples were excluded: (1) 16 males; (2) 15 questionnaires with a completion time of less than 60 s; (3) 3 participants aged younger than 18 years and 1 questionnaire with abnormal values; and (4) 42 questionnaires with incorrect responses to the trap question. Ultimately, 518 valid questionnaires were retained, with a validity rate of 87.06%. In the formal survey, the overall scale and its subscales demonstrated good internal consistency. The overall Cronbach’s α coefficient was 0.8462, while the coefficients for the knowledge, attitude, and practice subscales were 0.8683, 0.8012, and 0.7083, respectively. The Kaiser–Meyer–Olkin (KMO) value for the overall scale was 0.8474.

### Demographic information on participants

This cross-sectional study included 518 female patients with melasma. The majority were aged 31–50 years (67.0%), resided in urban areas (62.9%), and held a bachelor’s degree or above (48.8%). The cheeks were the most commonly reported location of melasma (61.4%), followed by the cheekbones (47.5%). The most frequently used treatments were topical whitening products (56.8%) and oral medications such as vitamin C or tranexamic acid (24.1%). Their mean (SD) knowledge, attitude, and practice scores were 8.92 (3.80), 22.97 (5.06), and 20.30 (3.88), respectively. Knowledge scores differed significantly by place of residence (*p* < 0.001), education level (*p* < 0.001), current employment status (*p* = 0.020), average daily sunlight exposure time (*p* = 0.011), regular use of sun protection measures (*p* < 0.001), average monthly income (*p* < 0.001), and whether patients had sought treatment for melasma (*p* < 0.001). Attitude scores varied significantly with the regular use of sun protection measures (*p* = 0.040). Practice scores showed significant associations with place of residence (*p* < 0.001), regular use of sun protection measures (*p* < 0.001), average monthly income (*p* < 0.001), and whether patients had sought treatment for melasma (*p* < 0.001) (Table 1).

**Table 1:** Demographic characteristics and KAP scores.

*N* = 518	*N* (%)	Knowledge mean (SD)	*P*	Attitude mean (SD)	*P*	Practice mean (SD)	*P*
Total score	518 (100.0)	8.92 (3.80)		22.97 (5.06)		20.30 (3.88)	
Age			0.898		0.085		0.923
<30	137 (26.4)	8.89 (3.46)		22.27 (5.67)		20.15 (4.21)	
31–40	158 (30.5)	8.85 (3.77)		23.71 (4.88)		20.25 (3.86)	
41–50	189 (36.5)	9.05 (3.89)		22.77 (4.47)		20.41 (3.77)	
≥50	34 (6.6)	8.56 (4.73)		23.47 (6.11)		20.50 (3.15)	
Place of residence			<0.001		0.787		<0.001
Urban	326 (62.9)	9.48 (3.67)		23.02 (4.68)		20.75 (3.81)	
Rural/Suburban	192 (37.1)	7.95 (3.82)		22.89 (5.67)		19.52 (3.87)	
Marital status			0.328		0.22		0.234
Married	373 (72.0)	8.82 (3.87)		23.14 (4.83)		20.17 (3.91)	
Single	145 (28.0)	9.18 (3.59)		22.53 (5.61)		20.62 (3.77)	
Education level			<0.001		0.851		0.066
Senior high school or below	161 (31.1)	7.92 (3.68)		22.91 (5.72)		19.96 (4.13)	
Associate degree	104 (20.1)	9.04 (3.60)		23.22 (5.06)		19.84 (3.93)	
Bachelor’s degree or above	253 (48.8)	9.50 (3.83)		22.90 (4.62)		20.70 (3.65)	
Current employment status			0.02		0.408		0.662
Office worker (indoor work)	337 (65.1)	9.33 (3.88)		22.72 (5.14)		20.45 (3.89)	
Worker with outdoor exposure	19 (3.7)	7.68 (2.94)		23.26 (5.08)		20.21 (4.17)	
Self-employed	106 (20.5)	8.16 (3.80)		23.32 (4.91)		20.08 (3.84)	
Homemaker	22 (4.2)	8.18 (2.74)		22.68 (3.68)		20.41 (3.25)	
Other	34 (6.6)	8.38 (3.46)		24.35 (5.50)		19.47 (4.08)	
Average daily sunlight exposure time			0.011		0.205		0.797
Less than 1 h	151 (29.2)	8.83 (3.81)		22.44 (5.36)		20.30 (4.26)	
1–2 h	225 (43.4)	9.00 (3.95)		23.39 (4.80)		20.45 (3.56)	
2–4 h	84 (16.2)	9.75 (3.40)		22.45 (4.99)		20.06 (4.04)	
More than 4 h	58 (11.2)	7.62 (3.39)		23.45 (5.31)		20.00 (3.84)	
Regularly use sun protection measures			<0.001		0.04		<0.001
Often	275 (53.1)	9.64 (3.75)		22.77 (5.04)		21.71 (3.54)	
Occasionally	207 (40.0)	8.57 (3.50)		22.87 (4.45)		19.05 (3.47)	
Never	36 (6.9)	5.39 (3.64)		25.03 (7.67)		16.61 (3.72)	
Average monthly income per capita			<0.001		0.074		<0.001
<2000	73 (14.1)	7.51 (3.88)		23.58 (5.24)		19.04 (3.61)	
2000–5,000	211 (40.7)	8.70 (3.64)		22.81 (5.41)		19.73 (3.81)	
5,000–10,000	164 (31.7)	9.41 (3.90)		22.41 (4.49)		21.13 (3.83)	
>10,000	70 (13.5)	9.89 (3.52)		24.13 (4.93)		21.34 (3.83)	
Duration of melasma			0.746		0.575		0.222
Less than 1 year	268 (51.7)	8.88 (3.62)		22.80 (4.97)		20.32 (3.78)	
1–3 years	90 (17.4)	9.19 (3.87)		22.84 (4.59)		20.83 (3.62)	
More than 3 years	160 (30.9)	8.82 (4.05)		23.32 (5.47)		19.95 (4.15)	
Sought treatment for melasma			<0.001		0.253		<0.001
No	427 (82.4)	8.57 (3.74)		23.09 (5.03)		19.84 (3.75)	
Yes	91 (17.6)	10.56 (3.63)		22.42 (5.20)		22.42 (3.75)	
Location of melasma (multiple choices)							
Cheekbones	246 (47.5)						
Forehead	82 (15.8)						
Cheeks	318 (61.4)						
Nose	59 (11.4)						
Perioral area	28 (5.4)						
Other	63 (12.2)						
Treatments for melasma you have used (multiple choices)							
Topical whitening products, such as creams containing vitamin C, arbutin, or similar ingredients	294 (56.8)						
Topical hydroquinone cream	26 (5.0)						
Oral medications such as vitamin C, vitamin E, tranexamic acid	125 (24.1)						
Laser therapy or mesotherapy (skin booster injections)	74 (14.3)						
Traditional Chinese medicine therapy	53 (10.2)						
Other	158 (30.5)						

### Distribution of responses to KAP

Responses to the knowledge dimension showed that the three questions with the lowest number of participants choosing the ‘Very familiar’ option were ‘Melasma is associated with genetic factors, and individuals with a family history of melasma are more likely to develop the condition.’ (K1) with 8.69%, ‘Light-based and laser therapies, such as intense pulsed light (IPL), picosecond lasers, and Q-switched lasers, can reduce pigmentation by targeting melanocytes, thereby achieving therapeutic effects for melasma.’ (K10) with 11.78%, and ‘Topical whitening products (such as creams containing vitamin C or arbutin), topical hydroquinone, and oral medications including vitamin C, vitamin E, and tranexamic acid can be used to treat melasma.’ (K9) with 14.86% (Supplementary Table S1).

Responses to the attitude dimension showed that 8.3% strongly agreed and 33.78% agreed that they feel uncomfortable or uneasy in public places because of melasma (A2), 8.3% strongly agreed and 31.27% agreed that they feel that they look unattractive because of melasma (A3), 6.76% strongly agreed and 21.24% agreed that the changes in their appearance caused by melasma was indirectly associated with their interpersonal relationships (A4) (Supplementary Table S2).

Responses to the practice dimension showed that only 3.28% always visit hospitals or medical aesthetics institutions to monitor the progression of melasma (P1), only 4.63% always wash their face more than three times a day (P4), only 5.02% always actively seek information related to melasma treatment and skin care through the internet, television, or other media (P6). However, 16.41% have never restricted their intake of tobacco, alcohol, coffee, very hot or cold foods, or spicy and greasy foods (P5) (Supplementary Table S3).

### Correlations between KAP

In the correlation analysis, significant positive correlations were found between knowledge and attitude (*r* = 0.145, *p* = 0.001), knowledge and practice (*r* = 0.390, *p* < 0.001), and attitude and practice (*r* = 0.223, *p* < 0.001), respectively (Table 2).

**Table 2:** Correlation analysis.

Spearman	Knowledge	Attitude	Practice
Knowledge	1.000		
Attitude	0.145 (*p* = 0.001)	1.000	
Practice	0.390 (*p* < 0.001)	0.223 (*p* < 0.001)	1.000

### Univariate and multivariate analysis of KAP

The median of the knowledge, attitude, and practice scores were used as the cut-off value for each dimension to divided the groups, and the number of participants above the cut-off value were 295 (56.9%), 293 (56.6%), and 300 (57.9%), respectively. Multivariate logistic regression showed that compared to urban residents, patients residing in rural or suburban areas had significantly lower knowledge (OR = 0.546, 95% CI: [0.363–0.820], *p* = 0.004). Patients who never used sun protection measures were less likely to have good knowledge compared to those who often did (OR = 0.384, 95% CI: [0.176–0.837], *p* = 0.016). Conversely, patients who had sought treatment for melasma demonstrated significantly higher knowledge (OR = 2.079, 95% CI: [1.230–3.515], *p* = 0.006). Meanwhile, a higher knowledge score was a significant independent predictor of a positive attitude (OR = 1.074, 95% CI: [1.022–1.129], *p* = 0.004). Compared to those with an average monthly per capita income of <2000 RMB, patients with an income between 2000 and 5,000 RMB had a lower likelihood of holding a positive attitude (OR = 0.512, 95% CI: [0.283–0.926], *p* = 0.027). Furthermore, higher knowledge (OR = 1.150, 95% CI: [1.081–1.223], *p* < 0.001) and attitude scores (OR = 1.071, 95% CI: [1.024–1.120], *p* < 0.001) were strong independent predictors of good practice. Regarding sun exposure, patients with 1–2 h of daily sunlight exposure were more likely to report good practice than those with less than 1 h (OR = 1.795, 95% CI: [1.106–2.913], *p* = 0.018). The regular use of sun protection was critically important: both occasional (OR = 0.264, 95% CI: [0.174–0.400], *p* < 0.001) and never use (OR = 0.157, 95% CI: [0.062–0.399], *p* < 0.001) were strongly associated with poorer practice compared to frequent use. Finally, patients who had sought treatment for melasma were more likely to have good practice (OR = 1.832, 95% CI: [1.014–3.310], *p* = 0.045) (Table 3).

**Table 3:** Univariate and multivariate logistic regression analysis.

Knowledge	Univariate analysis		Multivariate analysis	
	OR (95%CI)	*P*	OR (95%CI)	*P*
Age				
<30				
31–40	0.801 (0.503,1.272)	0.348		
41–50	0.894 (0.571,1.397)	0.624		
≥50	0.850 (0.399,1.834)	0.674		
Place of residence				
Urban				
Rural/Suburban	0.486 (0.338,0.698)	<0.001	0.546 (0.363,0.820)	0.004
Marital status				
Married				
Single	1.341 (0.908,1.993)	0.143		
Education level				
Senior high school or below				
Associate degree	1.333 (0.812,2.201)	0.258		
Bachelor’s degree or above	1.391 (0.934,2.074)	0.104		
Current employment status				
Office worker (indoor work)				
Worker with outdoor exposure	0.480 (0.182,1.217)	0.125	0.503 (0.185,1.365)	0.177
Self-employed	0.686 (0.442,1.064)	0.092	0.746 (0.459,1.214)	0.238
Homemaker	0.457 (0.184,1.089)	0.080	0.445 (0.172,1.149)	0.094
Other	1.066 (0.522,2.255)	0.862	1.381 (0.627,3.042)	0.422
Average daily sunlight exposure time				
Less than 1 h				
1–2 h	1.102 (0.726,1.672)	0.648	1.206 (0.778,1.872)	0.402
2–4 h	1.328 (0.771,2.309)	0.311	1.720 (0.951,3.109)	0.073
More than 4 h	0.588 (0.317,1.080)	0.089	0.817 (0.409,1.629)	0.565
Regularly use sun protection measures				
Often				
Occasionally	0.669 (0.463,0.964)	0.031	0.756 (0.514,1.111)	0.154
Never	0.295 (0.137,0.604)	0.001	0.384 (0.176,0.837)	0.016
Average monthly income per capita				
<2000				
2000–5,000	1.185 (0.695,2.023)	0.532	0.759 (0.422,1.364)	0.356
5,000–10,000	1.606 (0.921,2.807)	0.095	0.830 (0.442,1.558)	0.562
>10,000	1.970 (1.010,3.899)	0.049	0.933 (0.441,1.976)	0.856
Duration of melasma				
Less than 1 year				
1–3 years	0.832 (0.515,1.349)	0.454		
More than 3 years	0.851 (0.573,1.264)	0.423		
Sought treatment for melasma				
No				
Yes	2.141 (1.322,3.557)	0.003	2.079 (1.230,3.515)	0.006

Attitude	Univariate analysis		Multivariate analysis	
	OR (95%CI)	*P*	OR (95%CI)	*P*
Knowledge	1.074 (1.025,1.126)	0.003	1.074 (1.022,1.129)	0.004
Age				
<30				
31–40	1.518 (0.955,2.421)	0.078	1.452 (0.895,2.355)	0.131
41–50	1.187 (0.763,1.847)	0.446	1.094 (0.689,1.737)	0.704
≥50	1.046 (0.492,2.237)	0.907	0.913 (0.413,2.018)	0.822
Place of residence				
Urban				
Rural/Suburban	0.948 (0.662,1.359)	0.769		
Marital status				
Married				
Single	0.889 (0.605,1.310)	0.551		
Education level				
Senior high school or below				
Associate degree	0.983 (0.598,1.621)	0.947		
Bachelor’s degree or above	0.960 (0.643,1.429)	0.839		
Current employment status				
Office worker (indoor work)				
Worker with outdoor exposure	1.199 (0.474,3.169)	0.703	1.071 (0.405,2.834)	0.889
Self-employed	1.561 (0.999,2.467)	0.053	1.305 (0.802,2.124)	0.285
Homemaker	1.260 (0.529,3.128)	0.605	1.000 (0.391,2.559)	1.000
Other	1.409 (0.691,2.976)	0.353	1.265 (0.594,2.698)	0.542
Average daily sunlight exposure time				
Less than 1 h				
1–2 h	1.508 (0.995,2.289)	0.053	1.493 (0.968,2.304)	0.070
2–4 h	1.254 (0.734,2.150)	0.409	1.257 (0.714,2.212)	0.429
More than 4 h	1.398 (0.761,2.599)	0.283	1.038 (0.533,2.021)	0.912
Regularly use sun protection measures				
Often				
Occasionally	1.094 (0.761,1.576)	0.628		
Never	1.272 (0.631,2.642)	0.508		
Average monthly income per capita				
<2000				
2000–5,000	0.492 (0.276,0.855)	0.013	0.512 (0.283,0.926)	0.027
5,000–10,000	0.573 (0.317,1.017)	0.061	0.658 (0.354,1.223)	0.186
>10,000	0.733 (0.366,1.458)	0.376	0.821 (0.398,1.693)	0.594
Duration of melasma				
Less than 1 year				
1–3 years	1.161 (0.718,1.894)	0.545		
More than 3 years	1.097 (0.739,1.631)	0.646		
Sought treatment for melasma				
No				
Yes	0.745 (0.473,1.174)	0.203		

Practice	Univariate analysis		Multivariate analysis	
	OR (95%CI)	*P*	OR (95%CI)	*P*
Knowledge	1.210 (1.144, 1.278)	<0.001	1.150 (1.081,1.223)	<0.001
Attitude	1.075 (1.037, 1.115)	<0.001	1.071 (1.024, 1.120)	<0.001
Age				
<30				
31–40	1.354 (0.852,2.157)	0.201		
41–50	1.160 (0.745,1.807)	0.511		
≥50	1.216 (0.572,2.646)	0.614		
Place of residence				
Urban				
Rural/Suburban	0.640 (0.446,0.918)	0.015	0.986 (0.629,1.546)	0.951
Marital status				
Married				
Single	1.041 (0.707,1.540)	0.839		
Education level				
Senior high school or below				
Associate degree	0.910 (0.554,1.496)	0.708		
Bachelor’s degree or above	1.110 (0.743,1.657)	0.609		
Current employment status				
Office worker (indoor work)				
Worker with outdoor exposure	0.965 (0.381,2.552)	0.941		
Self-employed	0.916 (0.590,1.428)	0.696		
Homemaker	1.014 (0.426,2.519)	0.975		
Other	0.790 (0.389,1.618)	0.513		
Average daily sunlight exposure time				
Less than 1 h				
1–2 h	1.518 (0.999,2.311)	0.051	1.795 (1.106,2.913)	0.018
2–4 h	1.127 (0.660,1.934)	0.661	1.081 (0.581,2.011)	0.806
More than 4 h	1.019 (0.555,1.877)	0.952	1.859 (0.890,3.881)	0.099
Regularly use sun protection measures				
Often				
Occasionally	0.248 (0.167,0.363)	<0.001	0.264 (0.174,0.400)	<0.001
Never	0.098 (0.040,0.215)	<0.001	0.157 (0.062,0.399)	<0.001
Average monthly income per capita				
<2000				
2000–5,000	1.592 (0.933,2.741)	0.090	1.237 (0.657,2.329)	0.510
5,000–10,000	2.543 (1.452,4.503)	0.001	1.738 (0.884,3.416)	0.109
>10,000	2.956 (1.503,5.944)	0.002	1.773 (0.786,3.998)	0.168
Duration of melasma				
Less than 1 year				
1–3 years	1.453 (0.889,2.409)	0.141		
More than 3 years	0.957 (0.645,1.421)	0.826		
Sought treatment for melasma				
No				
Yes	3.069 (1.836,5.342)	<0.001	1.832 (1.014,3.310)	0.045

The median value was used as the cutoff for each dimension to dichotomize scores. For knowledge, 295 participants (56.9%) scored ≥9 and 223 (43.1%) scored ≤8. For attitude, 293 participants (56.6%) scored ≥23 and 225 (43.4%) scored ≤22. For practice, 300 participants (57.9%) scored ≥20 and 218 (42.1%) scored ≤19; ORs for knowledge and attitude scores represent the change in odds per one-point increase in the continuous score.

### Interactions between KAP

The structural equation model demonstrated acceptable fit (RMSEA = 0.062, SRMR = 0.069, TLI = 0.892, CFI = 0.904; Supplementary Table S4). In the path model, knowledge was directly associated with attitude (*β* = 0.153, *p* = 0.002) and practice (*β* = 0.492, *p* < 0.001), and attitude was directly associated with practice (*β* = 0.357, *p* < 0.001). An indirect association of knowledge with practice via attitude was also observed (*β* = 0.054, *p* = 0.002) (Table 4 and Figure 1).

**Table 4:** Standardized total, direct, and indirect path coefficients in the structural equation model.

Outcome	Predictor	Total effects		Direct Effect		Indirect effect	
		*β* (95%CI)	*P*	*β* (95%CI)	*P*	*β* (95%CI)	*P*
Attitude	Knowledge	0.153 (0.056, 0.249)	0.002	0.153 (0.056, 0.249)	0.002		
Practice	Knowledge	0.547 (0.467, 0.627)	<0.001	0.492 (0.412, 0.573)	<0.001	0.054 (0.019, 0.090)	0.002
	Attitude	0.357 (0.274, 0.439)	<0.001	0.357 (0.274, 0.439)	<0.001		

Dashes indicate no indirect path. β = standardized coefficient; CI = confidence interval. The indirect effect of knowledge on practice was mediated by attitude.

**Figure 1:**
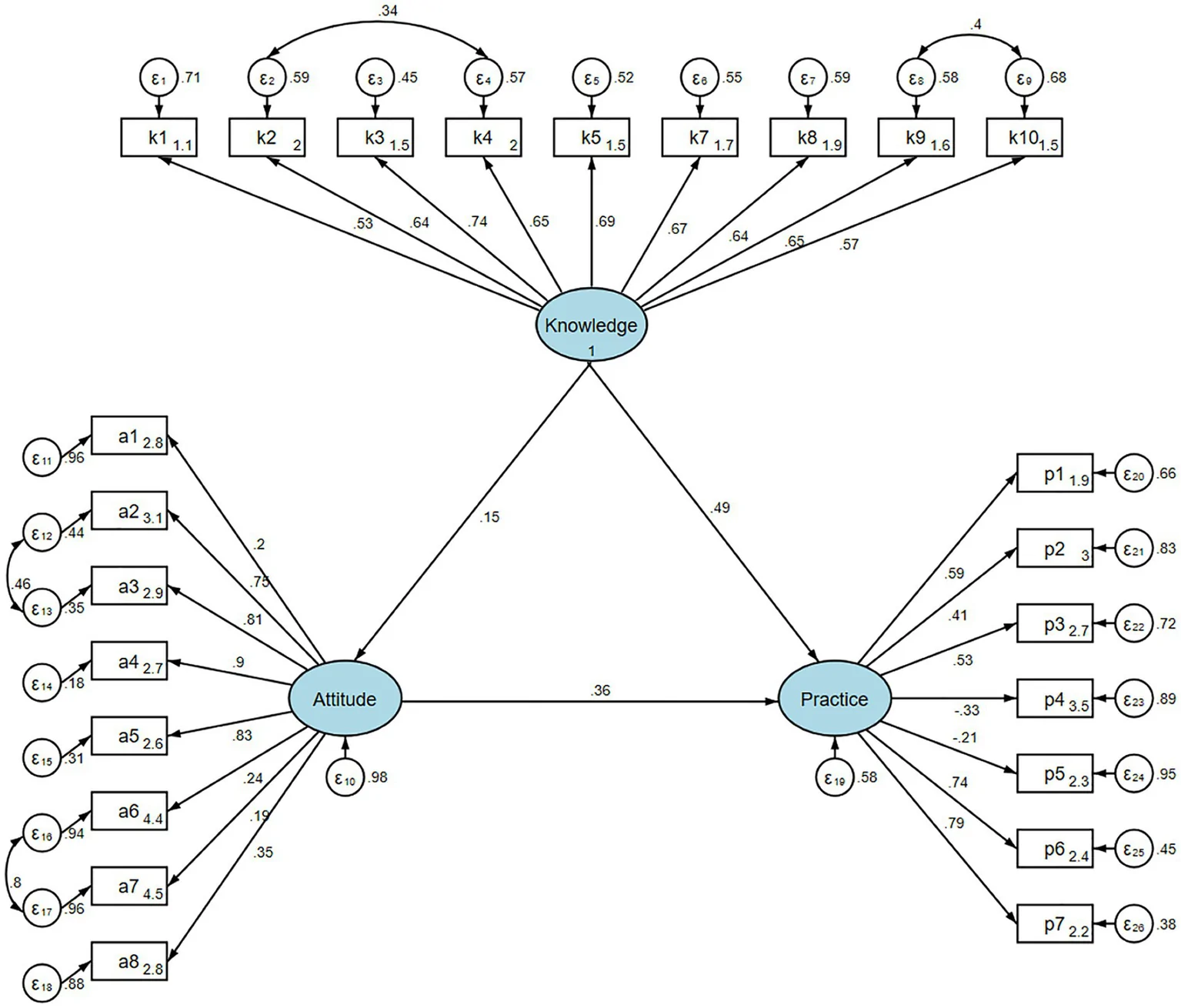
SEM model.

## Discussion

Knowledge scores were below the midpoint of the possible range (8.92 of 18), suggesting room for improvement and were positively linked to attitudes and self-management practices, with attitude partially mediating the association between knowledge and practice, indicating that addressing cognitive gaps may translate into behavioral gains. Implementing a brief, standardized melasma education package embedded into routine visits may feasibly improve adherence and sustain long-term disease control.

Item-level responses suggest that knowledge gaps concentrate in areas that patients often need when making everyday decisions about treatment choices and risk management (15). In the Chinese context, treatment choices may also be shaped by the structure of service access and commercial exposure. Qualitative and observational studies have shown that many Chinese patients with melasma first rely on over-the-counter whitening products, social media information, and medical aesthetics institutions rather than hospital-based dermatologic care, which may frame melasma predominantly as a cosmetic issue and contribute to fragmented knowledge about evidence-based management (9, 16, 17). Related research suggests that patient knowledge in chronic dermatologic conditions often develops through fragmented exposure to commercial messaging and informal advice, which can leave patients able to recognize terms without understanding how to apply them (18). KAP reporting guidance has emphasized that this kind of “recognition without functional use” can weaken the link between knowledge scores and meaningful behavior change, especially when knowledge items mix general beliefs with clinically actionable content (19). In melasma, reviews focused on photoprotection have argued that counseling should prioritize concepts that support daily decisions, such as what protection targets, how to use it correctly, and how it fits into maintenance therapy, rather than broad biomedical detail that patients cannot readily translate into routine action (15).

The observed differences in knowledge across residential settings point to information access and care pathways rather than to preference alone. Work on health literacy in China has described persistent regional heterogeneity tied to service availability, education opportunities, and the accessibility of credible health information (20). Evidence maps of teledermatology indicate that remote models can narrow access gaps in settings where specialist care concentrates in urban centers, but benefits depend on clear workflows and structured follow-up rather than on simple platform adoption (21). Melasma consensus documents have also treated counseling as an essential component of care because long-term control requires repeated reinforcement and support that many patients do not receive through occasional visits (16, 22). Together, these lines of evidence help frame why lower knowledge outside urban areas may coexist with limited monitoring and limited information-seeking behaviors, since both reflect constraints on sustained contact with standardized education (16).

Attitude responses indicate that melasma frequently affects how patients feel in social settings and how they perceive their appearance, which carries implications for adherence and care engagement (17). Related research suggests that quality-of-life impairment in melasma often extends beyond cosmetic concern and includes emotional distress and social avoidance, even when clinical severity appears modest (17). Prospective cross-sectional evidence has linked melasma with lower self-esteem and higher levels of anxiety-related symptoms, highlighting that psychological impact can remain substantial in sunny climates where exposure feels hard to avoid (23). More recent psychiatric-oriented observational work has also reported notable burdens of depressive and anxiety symptoms among people with melasma, supporting the clinical relevance of recognizing distress during routine dermatology encounters (5, 24). Consensus statements emphasize that disappointment after repeated treatment attempts can shape treatment beliefs and reduce persistence, which aligns with the presence of attitudinal items reflecting resignation or disengagement in some patients (16). The observation that patients with average monthly income between 2000 and 5,000 RMB showed less positive attitudes compared to those earning under 2000 RMB warrants careful interpretation. This pattern may reflect economic strain specific to lower-middle-income groups who face competing financial pressures—sufficient income to rule out public assistance programs but insufficient means to consistently afford cosmetic treatments or premium photoprotection products. Related socioeconomic research in China suggests that individuals in this income bracket often report heightened financial stress and deferred healthcare spending for non-acute conditions (25). Additionally, this group may hold higher expectations for aesthetic outcomes yet experience repeated disappointment when treatments prove unaffordable or ineffective, leading to more negative attitudes toward disease management compared to lower-income groups who may have adjusted expectations or higher-income groups with greater treatment access.

The associations among knowledge, attitudes, and practices suggest that behavioral change does not hinge on information alone, even when knowledge shows a clear link with practice (19). Related research suggests that attitudes often function as an interpretive filter, shaping whether patients treat advice as worth the effort, whether they anticipate benefit, and whether they persist when outcomes fluctuate (18). Patient education research in chronic skin diseases has noted that education tends to work best when it increases patients’ confidence in concrete tasks and clarifies expectations, rather than simply increasing factual recall (18). Melasma-focused photoprotection reviews likewise argue that counseling should address perceived burden and feasibility, including comfort, cosmetic acceptability, and how to handle imperfect adherence, because these factors influence whether patients sustain protection over time (15). Consensus guidance supports this approach by recommending that clinicians align expectations with the relapsing nature of melasma and emphasize maintenance, since unrealistic expectations can destabilize attitudes even when knowledge improves (16).

Practice distributions indicate limited routine monitoring and limited active information seeking, which can leave patients managing a chronic, relapse-prone condition without regular corrective feedback (16). The inclusion of face-washing frequency and dietary restriction items was intended to capture common patient behaviors; however, we acknowledge that the clinical relevance of exceeding three daily washes or avoiding coffee and spicy foods as melasma-specific management indicators is not strongly evidence-based. These items should be interpreted as descriptive of self-care patterns rather than as validated quality-of-practice metrics. The finding that patients with 1–2 h of daily sunlight exposure demonstrated better practice behaviors than those with less than 1 h appears counterintuitive, as greater sun exposure typically increases melasma risk. However, this association may reflect reverse causality or heightened awareness among individuals who experience more pronounced symptoms due to increased exposure. Patients who spend more time outdoors may develop more visible pigmentation changes, prompting them to seek professional guidance and adopt protective behaviors reactively rather than preventively. This interpretation aligns with the observation that treatment-seeking patients also showed significantly better practice scores, suggesting that symptom severity and personal experience of relapse may serve as stronger motivators for behavior change than abstract knowledge of risk factors alone. Related research suggests that when follow-up is infrequent, patients may respond to recurrence by switching products or pursuing unregulated interventions, which can increase risk and undermine trust in evidence-based care (15). Consensus documents highlight photoprotection as foundational and describe melasma management as long-term and iterative, implying that patients who do not build stable routines may cycle through short-lived improvements followed by relapse and discouragement (16). Evidence maps of teledermatology suggest that structured remote follow-up may help address this gap by enabling brief reinforcement, early troubleshooting of adherence barriers, and timely escalation when patients worsen, provided programs define responsibilities and maintain quality assurance (21). Health literacy work in China cautions that digital expansion can widen disparities if interventions assume uniform eHealth literacy, so follow-up systems need simple language and alternative pathways for those with lower digital capacity (20).

Photoprotection deserves special emphasis because contemporary evidence extends beyond ultraviolet radiation alone and includes the role of short-wavelength visible light in pigmentation pathways. Related research suggests that sunscreens formulated to protect against both ultraviolet and visible light can reduce relapse risk in melasma compared with ultraviolet-only protection, which supports counseling that goes beyond generic “broad-spectrum” advice (26). Controlled clinical work has compared visible light–protective tinted formulations with untinted products under real-world summer conditions and reported advantages for relapse prevention, reinforcing the practical value of recommending visible light–protective options when appropriate (27). These findings matter clinically because visible light protection often depends on formulation features such as iron oxides and tint, which can affect shade match and cosmetic acceptability, and these factors influence adherence in daily life (15). Consensus guidance has increasingly acknowledged the need to tailor photoprotection to patient context, including exposure patterns and product tolerability, rather than treating sunscreen choice as interchangeable across products (16).

The results support recommendations that stay close to the observed weaknesses in knowledge and practice and that can fit routine clinical workflows (18). First, clinics can implement a short standardized counseling script focused on daily photoprotection behaviors and maintenance principles, accompanied by a brief teach-back step to confirm understanding, since patient education reviews suggest that structured education improves outcomes when it targets self-management tasks and checks comprehension (18). Second, counseling can include concrete product guidance rather than general advice, including the option of visible light–protective tinted sunscreens when suitable, since trials and reviews indicate that protection characteristics and cosmetic acceptability both affect clinical outcomes and adherence (15). Third, patients who rarely use sun protection or who have lower access to care can receive low-burden digital reinforcement messages that prompt routine behaviors, since randomized trials in sun-protection behavior show that tailored, timely prompts can increase protective actions compared with usual information delivery (28). Fourth, services can pair digital reinforcement with a defined teledermatology pathway for troubleshooting and escalation, since evidence mapping suggests that remote dermatology works best when integrated into care pathways with clear triage criteria and accountability (21).

Attitude-related findings justify more explicit attention to psychosocial burden during melasma management rather than treating distress as incidental (17). Related research suggests that quality-of-life impairment in melasma can persist even when patients pursue treatment, indicating that symptom control and emotional well-being do not always move in parallel (17). Observational evidence has linked melasma with anxiety and depressive symptoms, supporting brief screening questions in dermatology settings and referral options when distress interferes with functioning or adherence (23). In practice, clinicians can normalize the relapsing course, address treatment fatigue, and frame maintenance as a controllable routine rather than as a sign of failure, consistent with consensus recommendations that emphasize expectation management and long-term planning (16). Where staffing allows, nurse-led follow-up can check barriers to photoprotection, discuss cosmetic concerns around tinted products, and reinforce safe treatment pathways, which aligns with patient education evidence supporting team-based reinforcement for chronic skin disease management (18).

Several limitations of this study should be acknowledged. First, the cross-sectional design precludes causal inference among knowledge, attitudes, and practices. The observed associations, particularly the mediation effects identified in the structural equation modeling, should therefore be strictly interpreted as correlational rather than causal, despite the use of multivariate analyses to control for potential confounders. Second, the use of convenience sampling and an online, self-administered questionnaire may have introduced selection and reporting biases, potentially overrepresenting participants with higher health awareness, digital literacy, or interest in melasma, which may limit the representativeness and external generalizability of the findings. Third, KAP data were based on self-reported measures, which are inherently subject to recall and social desirability bias, although questionnaire quality was strengthened through expert review, pilot testing, and internal consistency assessment.

## Conclusion

In summary, this study found that knowledge, attitudes, and practices regarding melasma among female patients indicated areas for improvement, particularly in photoprotection adherence and routine monitoring, and higher knowledge was associated with better practice both directly and indirectly through more favorable attitudes. Clinically, implementing a structured, photoprotection-centered education pathway combining brief standardized counseling at visits, tailored materials for lower-knowledge groups, and low-cost digital follow-up to reinforce key behaviors may pragmatically improve adherence and long-term self-management.

## Data Availability

The original contributions presented in the study are included in the article/Supplementary material, further inquiries can be directed to the corresponding author/s.
