# Supplementary material for: Knowledge, attitudes, and practices regarding melasma among female patients
Source: Front Public Health. 2026 Sep 18;14:1907193. doi: 10.3389/fpubh.2026.1907193 (PMC13630645; doi:10.3389/fpubh.2026.1907193)
Supplement: Supplementary file 1 [file Table_1.DOCX]

**Table S1. Distribution of knowledge dimension responses**

| **Knowledge** | **Very familiar** | **Heard of it** | **Not sure** |
| --- | --- | --- | --- |
| 1. Melasma is associated with genetic factors, and individuals with a family history of melasma are more likely to develop the condition. | 45 (8.69%) | 254 (49.03%) | 219 (42.28%) |
| 2. Long-term exposure to sunlight can cause skin hyperpigmentation, which may lead to the development of melasma. | 136 (26.25%) | 332 (64.09%) | 50 (9.65%) |
| 3. Physiological processes such as pregnancy, use of oral contraceptives, and menopause may lead to hormonal changes, which can trigger melasma. | 89 (17.18%) | 309 (59.65%) | 120 (23.17%) |
| 4. Unhealthy lifestyle habits, such as staying up late, irregular diet, excessive stress, as well as improper skin care practices (e.g., using skincare products containing excessive levels of lead or mercury), may also contribute to the development of melasma. | 140 (27.03%) | 329 (63.51%) | 49 (9.46%) |
| 5. Certain systemic diseases, such as thyroid disorders, female reproductive system diseases, and liver diseases, have been reported to be associated with the occurrence of melasma. | 75 (14.48%) | 326 (62.93%) | 117 (22.59%) |
| 7. Maintaining a positive mood can promote blood circulation and metabolism, which may help prevent the development of melasma. | 124 (23.94%) | 309 (59.65%) | 85 (16.41%) |
| 8. Avoiding skincare products containing irritating ingredients and avoiding excessive rubbing of the face can help protect the skin barrier. | 141 (27.22%) | 315 (60.81%) | 62 (11.97%) |
| 9. Topical whitening products (such as creams containing vitamin C or arbutin), topical hydroquinone, and oral medications including vitamin C, vitamin E, and tranexamic acid can be used to treat melasma. | 77 (14.86%) | 330 (63.71%) | 111 (21.43%) |
| 10. Light-based and laser therapies, such as intense pulsed light (IPL), picosecond lasers, and Q-switched lasers, can reduce pigmentation by targeting melanocytes, thereby achieving therapeutic effects for melasma. | 61 (11.78%) | 339 (65.44%) | 118 (22.78%) |

Note: Data are presented as n (%).

**Table S2. Distribution of attitude dimension responses**

| **Attitude** | **Strongly agree** | **Agree** | **Neutral** | **Disagree** | **Strongly disagree** |
| --- | --- | --- | --- | --- | --- |
| 1. I believe that melasma is a natural sign of aging and does not require treatment. | 16 (3.09%) | 59 (11.39%) | 138 (26.64%) | 272 (52.51%) | 33 (6.37%) |
| 2. Because of melasma, I feel uncomfortable or uneasy in public places. | 43 (8.3%) | 175 (33.78%) | 170 (32.82%) | 103 (19.88%) | 27 (5.21%) |
| 3. Because of melasma, I feel that I look unattractive. | 43 (8.3%) | 162 (31.27%) | 160 (30.89%) | 117 (22.59%) | 36 (6.95%) |
| 4. The changes in my appearance caused by melasma have affected my interpersonal relationships. | 35 (6.76%) | 110 (21.24%) | 158 (30.5%) | 171 (33.01%) | 44 (8.49%) |
| 5. I worry that people may judge me based on the melasma on my face, such as assuming I have unhealthy eating habits or poor hygiene. | 25 (4.83%) | 120 (23.17%) | 124 (23.94%) | 207 (39.96%) | 42 (8.11%) |
| 6. Compared with medical aesthetics institutions, I believe that hospitals provide more standardized diagnosis and treatment. | 126 (24.32%) | 257 (49.61%) | 98 (18.92%) | 29 (5.6%) | 8 (1.54%) |
| 7. Compared with medical aesthetics institutions, I believe that physicians in hospitals are more trustworthy. | 137 (26.45%) | 265 (51.16%) | 82 (15.83%) | 26 (5.02%) | 8 (1.54%) |
| 8. Because multiple treatment attempts have been ineffective, I have given up treating my melasma. | 23 (4.44%) | 83 (16.02%) | 175 (33.78%) | 202 (39%) | 35 (6.76%) |

Note: Data are presented as n (%).

**Table S3. Distribution of practice dimension responses**

| **Practice** | **Always** | **Often** | **Sometimes** | **Rarely** | **Never** |
| --- | --- | --- | --- | --- | --- |
| 1. I regularly visit hospitals or medical aesthetics institutions to monitor the progression of melasma. (P) | 17 (3.28%) | 35 (6.76%) | 69 (13.32%) | 186 (35.91%) | 211 (40.73%) |
| 2. I pay attention to sun protection in daily life and avoid exposure to strong ultraviolet radiation. (P) | 120 (23.17%) | 142 (27.41%) | 139 (26.83%) | 90 (17.37%) | 27 (5.21%) |
| 3. I consistently use moisturizing and anti-pigmentation skincare products. (P) | 78 (15.06%) | 142 (27.41%) | 137 (26.45%) | 116 (22.39%) | 45 (8.69%) |
| 4. I wash my face more than three times a day. (N) | 24 (4.63%) | 47 (9.07%) | 129 (24.9%) | 213 (41.12%) | 105 (20.27%) |
| 5. I have never restricted my intake of tobacco, alcohol, coffee, very hot or cold foods, or spicy and greasy foods. (N) | 85 (16.41%) | 108 (20.85%) | 137 (26.45%) | 116 (22.39%) | 72 (13.9%) |
| 6. I actively seek information related to melasma treatment and skin care through the internet, television, or other media. (P) | 26 (5.02%) | 87 (16.8%) | 142 (27.41%) | 185 (35.71%) | 78 (15.06%) |
| 7. I actively communicate with other patients about skincare experiences and products. (P) | 25 (4.83%) | 74 (14.29%) | 135 (26.06%) | 180 (34.75%) | 104 (20.08%) |

Note: Data are presented as n (%).

**Table S4. SEM fit indicators**

| **Indicators** | **Reference** | **Results** |
| --- | --- | --- |
| RMSEA | <0.080 | 0.062 |
| SRMR | <0.080 | 0.069 |
| TLI | >0.800 | 0.892 |
| CFI | >0.800 | 0.904 |
